# Full-endoscopic posterior longitudinal ligament coverage suturing for lumbar disc herniation with annular defects

**DOI:** 10.1016/j.jor.2025.03.048

**Published:** 2025-03-26

**Authors:** Quan Zhang, Bo He, Zijin Liu, Zhenfei Wang, Jinhui Bu, Minghui Dong, Zhaobo Liu, Mengzi Hu, Chao Ma, Guangwang Liu

**Affiliations:** a Xuzhou Clinical School of Xuzhou Medical University, Department of Orthopedic Surgery, Xuzhou Central Hospital, Xuzhou, Jiangsu, 221009, China; b Xuzhou No. 1 Middle School, Xuzhou, Jiangsu, 221002, China; c Xuzhou Clinical School of Xuzhou Medical University, Department of Orthopedic Surgery, Xuzhou Central Hospital, Xuzhou Central Hospital Affiliated to Medical School of Southeast University, Xuzhou School of Clinical Medicine of Nanjing Medical University, Xuzhou Central Hospital Affiliated to Nanjing University of Chinese Medicine, Xuzhou, Jiangsu, 221009, China; d Department of Spine Surgery, Changsha Central Hospital, University of South China, Changsha, Hunan, 410004, China; e Department of Orthopedics, Affiliated Hangzhou First People's Hospital Chengbei Campus, School of Medicine, Westlake University, Hangzhou, Zhejiang Province, 310000, China

**Keywords:** Annulus fibrosus, Posterior longitudinal ligament, Lumbar disc herniation

## Abstract

**Background:**

To investigate the clinical efficacy of full-endoscopic posterior longitudinal ligament (PLL) coverage suturing in the area of the defective annulus fibrosus for the treatment of lumbar disc herniation (LDH).

**Methods:**

Retrospective analysis of patients who underwent full-endoscopic surgery for LDH in our hospital from March 2018 to July 2023, there were 56 cases presented with partial defects of the annulus fibrosus detected microscopically. Among these, 34 cases were treated with full-endoscopic lumbar discectomy alone (control group), whereas 22 cases were treated with posterior longitudinal ligament coverage of annulus fibrosus defects with suturing following lumbar discectomy (observation group). Perioperative, follow-up and imaging data were compared between the two groups.

**Results:**

No severe complications occurred in either group. The observation group had longer surgical time (67.23 ± 8.85 vs. 54.38 ± 8.16 min, *P* < 0.05), but no differences in incision length, blood loss, or hospital stay (*P* > 0.05). At 12–18-month follow-up (mean 14.3 ± 0.2 months), recurrence was observed in 6 patients (17.6 %) in the control group versus none in the observation group (*P* < 0.05); one recurrence required reoperation, with no intergroup difference in reoperation rates (*P* > 0.05). Preoperative VAS and ODI scores were comparable (*P* > 0.05), and both groups showed significant postoperative reductions (*P* < 0.05), though intergroup differences were insignificant (*P* > 0.05). Pfirrmann grading improved significantly in both groups postoperatively (*P* < 0.05) but did not differ between groups (*P* > 0.05). Full-endoscopic posterior longitudinal ligament coverage suturing effectively repaired annular defects, reduced recurrence, and demonstrated clinical efficacy for lumbar disc herniation.

**Conclusions:**

Lumbar disc herniation with annulus fibrosus defects can be treated with satisfactory clinical efficacy by using full-endoscopic posterior longitudinal ligament coverage suturing in the area of the defective annulus fibrosus, which can effectively repair fibrous annulus defects and reduce the recurrence rate of LDH.

## 1 Background

Lumbar disc herniation (LDH) is a common spinal condition that is particularly prevalent among young to middle-aged men who are engaged in heavy physical labor. It primarily manifests as lower back pain, radiculopathy (pain radiating along the nerve root path, typically down the leg), and sensory abnormalities, significantly impacting patients' quality of life and resulting in economic and psychological burdens for both individuals and their families.

For patients with LDH, conservative management, including bed rest, oral nonsteroidal anti-inflammatory drugs (NSAIDs), physical therapy, and massage, is typically initiated for 6–12 weeks. Patients whose symptoms do not improve following conservative treatment may ultimately require surgical intervention.^1^ Full-endoscopic lumbar discectomy is currently one of the mainstream surgical approaches for treating lumbar disc herniation. However, the reoperation rate for this procedure can be as high as 8 %–15 %.^2^, ^3^, ^4^ Reducing the recurrence rate and reoperation rate has been a focal point of concern for spinal surgeons. Addressing these issues is crucial for improving patient outcomes and long-term success rates. Although suturing the annulus fibrosus can promote scar healing at the site of rupture and help reduce the recurrence rate, the mechanical strength of the resulting scar tissue remains inferior to that of a normal, intact annulus fibrosus.^5^ Moreover, for larger defects, the surrounding weakened annulus fibrosus cannot adequately withstand the tension generated at the defect site, making intraoperative suturing challenging and significantly reducing the effectiveness of the surgery. Therefore, addressing the repair of larger annulus fibrosus defects remains a critical clinical challenge.

The posterior longitudinal ligament (PLL) is located posterior to the vertebral bodies and is connected to the upper and lower margins of the vertebral bodies and the intervening annulus fibrosus. Its primary functions are to protect the spinal cord and nerve roots from damage caused by herniated discs and to limit and support spinal movement, thereby maintaining spinal stability.^6^ During endoscopic procedures, we observed that the PLL covered the posterior aspect of the vertebral body and the surface of the annulus fibrosus, which was loosely connected to the vertebral body and the annulus fibrosus, and it was easy to mobilize and dissect intraoperatively, with good space for transposition. Building on the foundation of full-endoscopic lumbar discectomy for LDH, we moderately loosened the PLL, transposed the PLL to cover the area of the annulus fibrosus defect and sutured it to the residual annulus fibrosus for effective repair of the annulus fibrosus defect. We conducted a retrospective analysis to evaluate the safety and efficacy of full-endoscopic lumbar discectomy combined with PLL coverage and suturing for repairing annular defects in patients with lumbar disc herniation.

## 2 Materials and methods

### 2.1 Subjects

We conducted a retrospective analysis of 22 patients with lumbar disc herniation who underwent full-endoscopic lumbar discectomy combined with posterior longitudinal ligament coverage suturing in the area of the defective annulus fibrosus at Xuzhou Central Hospital between March 2018 and July 2023 (observation group). The observation group comprised 14 males and 8 females, with a mean age of 41.8 ± 10.6 years (range: 18–62 years). For comparison, we selected 34 LDH patients who were treated with full-endoscopic lumbar discectomy alone during the same period (control group), including 22 males and 12 females, with a mean age of 43.4 ± 9.8 years (range: 17–59 years). There were no significant differences between the two groups in terms of age, gender, body mass index (BMI), course of disease, or responsible levels (P > 0.05) (Table 1). The study protocol was approved by the Ethics Review Committee of Xuzhou Central Hospital. All patients were informed about the study, and written informed consent was obtained from each participant.

**Table 1:** Comparison of general data between the two groups before treatment.

Indicator	Control Group (n = 34)	Observation Group (n = 22)	Statistical Value	P-value
Age (years)	43.4 ± 9.8	41.8 ± 10.6	*t* = 0.605	0.548
Gender (male/female)	22/12	14/8	*X*^2^ = 0.007	0.935
BMI(kg/m^2^)	22.7 ± 2.6	23.1 ± 2.8	*t* = −0.555	0.581
Course of disease (months)	13.0 ± 2.6	13.4 ± 2.4	*t* = −0.546	0.587
Responsible levels (L_3/4_/L_4/5_/L_5_S_1_)	8/14/12	5/9/8	*X*^2^ = 0.008	0.996

The inclusion criteria were as follows: (1) Patients who were diagnosed with single-level lumbar disc herniation on the basis of CT, MRI findings, clinical symptoms, and physical examination. (2) Patients who had undergone at least 3 months of strict conservative treatment without improvement and who showed a trend toward worsening symptoms. (3) X-ray images in flexion–extension views of the lumbar spine demonstrated no significant instability. (4) Endoscopic observation revealed partial tearing of the annulus fibrosus from the posterior margin of the vertebral body, with an annular defect width ≥6 mm. (5) Patients who underwent either full-endoscopic lumbar discectomy alone or full-endoscopic lumbar discectomy combined with PLL coverage and annular defect suturing, with signed informed consent.

The exclusion criteria were as follows: (1) Significant calcification around the annulus fibrosus or severe ossification of the posterior longitudinal ligament. (2) History of previous lumbar surgery, tumor, infection, or other relevant conditions. (3) Presence of severe systemic diseases or conditions that do not meet surgical criteria. (4) Cognitive impairment or concurrent psychiatric disorders that hinder effective cooperation with treatment and follow-up. (5) Incomplete clinical data.

### 2.2 Surgical procedure

All surgeries were performed under local anesthesia (2 % lidocaine diluted to 10–30 ml), with patients in the prone position and their abdomen suspended. The target intervertebral space was localized via a C-arm X-ray machine, followed by standard disinfection and draping.

Control group: A 1 cm skin incision was made 1–1.5 cm lateral to the spinous process. Sharp dissection through the deep fascia allowed the insertion of a conical dilator into the dorsal aspect of the ligamentum flavum between the laminae. C-arm fluoroscopy confirmed the position before placing a 7.0 mm working cannula and inserting the endoscope connected to a radiofrequency ablation system. The fibrofatty tissue on the surface of the ligamentum flavum was cleared via nucleus pulposus forceps, and radiofrequency ablation was applied for hemostasis, which fully exposed the ligamentum flavum. A laminectomy forceps was then used to resect part of the ligamentum flavum, allowing access to the spinal canal and fully exposing the dura mater and nerve roots. The working cannula was carefully inserted and rotated to protect the nerve roots. The herniated nucleus pulposus was extracted via nucleus pulposus forceps, and the torn annulus fibrosus was partially removed while preserving its attachment. Radiofrequency ablation was applied around the annular tear for remodeling. After adequate neural decompression was confirmed, the working channel was withdrawn, a negative pressure drain was placed, and the incision was closed layer by layer and dressed with sterile gauze.

**Fig. 1:**
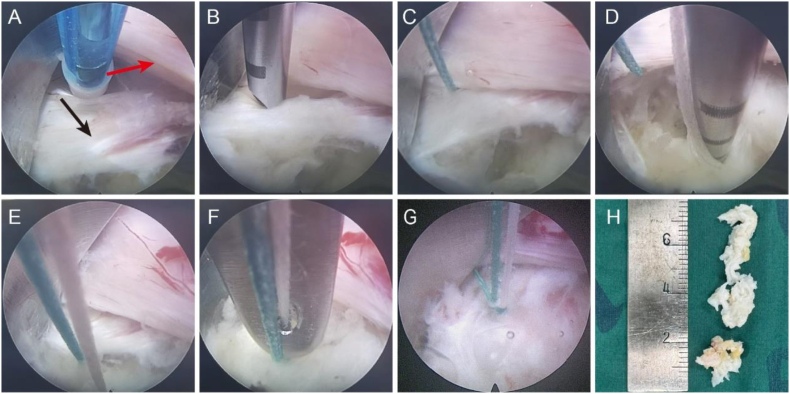
Intraoperative visualization of the suturing process for PLL coverage of annulus fibrosus defects. (A) The posterior longitudinal ligament was repositioned to cover the area of the annulus fibrosus defect (red arrow pointing to the nerve root, black arrow pointing to the PLL). (B) The annulus fibrosus suture device was sequentially passed through the PLL and the annulus fibrosus. (C–E) The PLL and the annulus fibrosus were sutured together. (F, G) The knots were tied using an endoscopic knot pusher. (H) The excised nucleus pulposus.

Observation group: The nucleus pulposus removal technique was identical to that described above. The PLL was mobilized to achieve sufficient laxity and then transposed to cover the annulus fibrosus defect. A disposable annulus fibrosus suture device was used to sequentially pass through the PLL and approximately 2 mm from the edge of the annulus fibrosus tear. An anchor-loaded internal screw was inserted via a guidewire, after which the needle and guidewire were removed. The same method was applied to suture the opposite side, ensuring complete coverage of the annulus fibrosus defect by the PLL. The knots were tied via an endoscopic knot pusher, and excess suture material was trimmed under endoscopic guidance (Fig. 1). After adequate neural decompression was confirmed, the working channel was withdrawn, a negative pressure drain was placed, and the incision was closed layer by layer and dressed with sterile gauze.

Postoperative management included routine antimicrobial, anti-inflammatory, analgesic, and neurotrophic treatments. The drainage tubes were removed 24–48 h after surgery, and patients were encouraged to ambulate with a lumbar brace starting 3 days after surgery.

### 2.3 Evaluation index

Data on surgical time, incision length, intraoperative blood loss, and hospital stay were collected and recorded for both groups. Pain intensity was assessed via the Visual Analog Scale (VAS), and functional disability was evaluated via the Oswestry Disability Index (ODI) preoperatively, 3 days after surgery, 3 months after surgery, and at the final follow-up to quantify and evaluate the effectiveness of the treatment. Imaging studies were performed, and lumbar disc degeneration was graded via the Pfirrmann grading system. Postoperative complications, LDH recurrence, and reoperation were also recorded.

### 2.4 Statistical analysis

Statistical analysis was performed via SPSS 26.0 software. Continuous data are presented as the mean ± standard deviation (‾χ±s). Intergroup comparisons were conducted via independent samples t tests, whereas intragroup comparisons at two time points were analyzed via paired t tests. Categorical data were analyzed via chi-square tests or Fisher's exact tests where appropriate. For ordinal data, intergroup comparisons were made via the Mann‒Whitney *U* test, and intragroup changes were assessed via the Wilcoxon signed‒rank test. P < 0.05 was considered statistically significant for all analyses.

## 3 Results

### 3.1 Perioperative outcomes

All patients in both groups successfully completed the surgery without experiencing any major complications, such as nerve root injury, cerebrospinal fluid leakage, or infection, during the procedure. The surgical time was significantly longer in the observation group than in the control group [(67.23 ± 8.85) min vs (54.38 ± 8.16) min, P < 0.05]. However, there were no statistically significant differences between the two groups in terms of incision length, intraoperative blood loss, or hospital stay (*P* > 0.05) (Table 2).

**Table 2:** Comparison of the perioperative data between the two groups. (‾χ±s).

Indicator	Control Group (n = 34)	Observation Group (n = 22)	Statistical Value	P-value
Surgical time (min)	54.38 ± 8.16	67.23 ± 8.85	*t* = −5.567	<0.001
Incision length (cm)	1.27 ± 0.20	1.22 ± 0.18	*t* = 1.088	0.282
Intraoperative blood loss (ml)	18.06 ± 3.81	19.18 ± 4.06	*t* = −1.050	0.298
Hospital stay (days)	7.47 ± 1.78	7.05 ± 1.50	*t* = 0.928	0.358

### 3.2 Comparison of follow-up results

All patients completed follow-up, with durations ranging from 12 to 18 months (mean: 14.3 ± 0.2 months). During the follow-up period, no cases of recurrence were observed in the observation group. In the control group, 6 patients (17.6 %) experienced recurrence, with 1 patient requiring reoperation after ineffective conservative treatment. The recurrence rate in the control group was significantly higher than that in the observation group (P < 0.05). However, there was no statistically significant difference in reoperation rates between the two groups (P > 0.05) (Table 3). Preoperatively, there were no statistically significant differences in the VAS or ODI scores between the observation and control groups (P > 0.05). Over time, both groups showed significant reductions in low back pain VAS, leg pain VAS, and ODI scores (P < 0.05). At the corresponding time points, comparisons of the VAS and ODI scores between the observation and control groups did not reveal any statistically significant differences (P > 0.05) (Table 4).

**Table 3:** Comparison of postoperative recurrence and reoperation between the two groups.

Group	n	Recurrence	Reoperation
Control group	34	6	1
Observation group	22	0	0
*X*^2^		4.348	0.659
*P*		0.037	0.417

**Table 4:** Comparison of follow-up data between the two groups (‾χ±*s*).

Indicator	Control Group (n = 34)	Observation Group (n = 22)	T Value	P value
**Low Back Pain VAS Score**				
Preoperative	7.06 ± 1.65	6.86 ± 1.83	0.414	0.681
3 days after surgery	2.59 ± 0.70^a^	2.77 ± 0.87^a^	−0.875	0.386
3 months after surgery	1.91 ± 0.93^a^	1.82 ± 0.59^a^	0.419	0.677
final follow-up	1.56 ± 0.96^a^	1.41 ± 0.59^a^	0.655	0.515
**Leg Pain VAS Score**				
Preoperative	6.62 ± 2.08	6.86 ± 1.91	−0.447	0.657
3 days after surgery	2.47 ± 0.66^a^	2.55 ± 0.80^a^	−0.380	0.705
3 months after surgery	1.68 ± 0.98^a^	1.64 ± 0.73^a^	0.165	0.869
final follow-up	1.41 ± 1.26^a^	1.18 ± 0.80^a^	0.763	0.449
**ODI index**				
Preoperative	64.82 ± 7.40	68.27 ± 8.54	−1.603	0.115
3 days after surgery	18.00 ± 3.65^a^	17.91 ± 3.78^a^	0.090	0.929
3 months after surgery	6.47 ± 4.80^a^	6.27 ± 3.04^a^	0.172	0.864
final follow-up	4.35 ± 4.28^a^	3.18 ± 1.18^a^	1.250	0.217

Note:

a Compared with preoperative values, P < 0.05.

### 3.3 Imaging evaluation

**Fig. 2:**
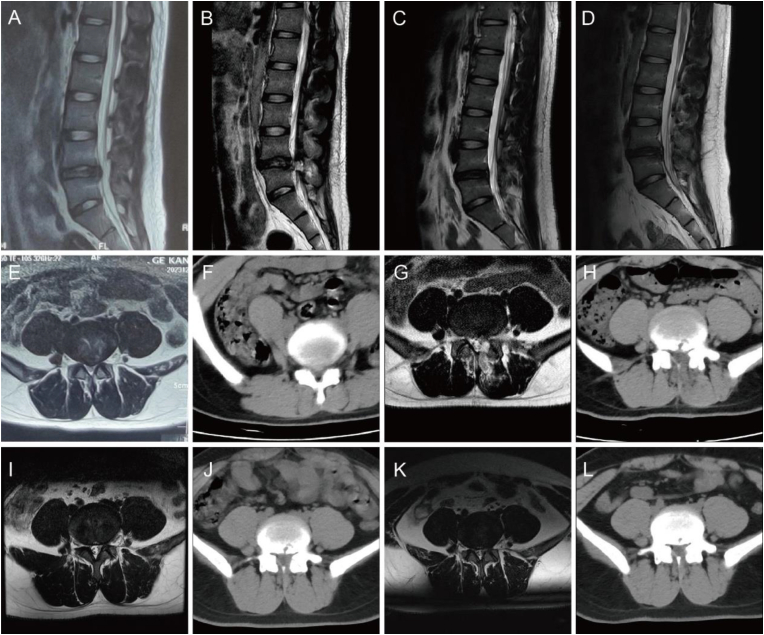
MRI and CT images of a 31-year-old male patient preoperatively and 3 days, 3 months, and 1 year postoperatively. (A, E, F) Preoperative sagittal MRI, axial MRI and CT revealed lumbar disc herniation at the L4/5 level. (B, G, H) Sagittal MRI, axial MRI and CT images at 3 days postoperatively. (C, I, J) At 3 months postoperatively, sagittal MRI, axial MRI and CT scans revealed no evidence of nucleus pulposus protrusion, with good repair of the annulus fibrosus. (D, K, L) At 1 year postoperatively, sagittal MRI, axial MRI, and CT scans showed no significant differences compared to the findings at three months postoperatively.

Compared with the preoperative values, the Pfirrmann grading significantly decreased at the final follow-up in both groups (P < 0.05). Preoperatively and at the final follow-up, there were no statistically significant differences in the Pfirrmann grading between the control group and the observation group (P > 0.05) (Table 5) (Fig. 2).

**Table 5:** Comparison of the Pfirrmann grading between the two groups.

Indicator	Control Group (n = 34)				Observation Group (n = 22)				P-value
Pfirrmann Grading	I	II	III	IV	I	II	III	IV	
Preoperative	0	0	21	13	0	0	15	7	0.628
final follow-up	5	7	15	7	3	5	9	5	0.944
P-value	<0.001				0.004

## 4 Discussion

The intervertebral disc is a crucial component of the spine and is characterized as an avascular structure primarily composed of three parts: the outer annulus fibrosus, the central nucleus pulposus, and the cartilaginous endplates at the upper and lower ends.^7^ Its primary functions include cushioning the pressure exerted on the spine, maintaining spinal stability and flexibility, and protecting the spinal cord and nerve roots. Degenerative disc disease is the pathological basis for LDH.^8^ With increasing age, the discs lose water content, leading to decreased elasticity of the nucleus pulposus and the development of fissures or tears in the annulus fibrosus. When combined with repetitive physical strain, these degenerative changes can result in posterior protrusion or extrusion of the nucleus pulposus through the weakened annulus fibrosus. In severe cases, this displacement can compress adjacent nerve roots, resulting in symptomatic manifestations.^9^

Currently, domestic and international scholars have largely reached a consensus on the treatment principles and surgical indications for LDH. However, the choice of surgical method remains a topic of active discussion among spinal surgeons. Although traditional open surgery has evolved over several decades to become highly mature, its significant drawbacks, such as substantial soft tissue damage and prolonged recovery times, can negatively impact patient outcomes. In recent years, with the rapid development of minimally invasive techniques and continuous advancements in endoscopic tools, endoscopic treatment of LDH has emerged as a safe and effective approach that is widely accepted. Percutaneous endoscopic lumbar discectomy (PELD) can be categorized into different approaches, including percutaneous endoscopic interlaminar discectomy (PEID) and percutaneous endoscopic transforaminal discectomy (PETD), on the basis of the surgical entry route used.^10^ Zhang et al.^11^ conducted a meta-analysis and reported that PETD is as effective as open lumbar discectomy (OLD) in treating LDH, but it offers advantages such as a lower complication rate, shorter incision length, and shorter hospital stay, which are typical benefits of minimally invasive surgery. Li et al.^12^ compared the effectiveness of PEID and PETD in treating LDH and found that both techniques are safe, effective, minimally invasive, and associated with minimal blood loss. However, PEID demonstrated superiority over PETD in terms of a shorter surgical time and fewer intraoperative fluoroscopy sessions.

The primary function of the annulus fibrosus is to protect the nucleus pulposus and maintain the shape and function of the intervertebral disc. In patients with LDH and associated annulus fibrosus defects, the loss of structural integrity at the defective site increases the risk of recurrent nucleus pulposus protrusion postsurgery, potentially necessitating further surgical intervention for severe symptoms. In recent years, annulus fibrosus repair techniques have emerged as a focus of research and clinical practice among spinal surgeons. Previous studies have shown that, compared with conventional discectomy, full-endoscopic discectomy combined with annulus fibrosus repair is a safe and effective minimally invasive technique that can effectively lower the recurrence rate of LDH.^13^ This innovative approach not only enhances surgical efficacy but also minimizes postoperative complications. The development and application of this technology represent a substantial leap forward in the minimally invasive management of LDH, offering patients improved long-term results and a reduced risk of recurrence.

Studies have shown a close association between the degree of annulus fibrosus defects and the recurrence rate following discectomy in patients with LDH.^14^ For patients with larger annulus fibrosus defects, inner annulus fibrosus injury is more severe, leading to limited healing potential and a higher risk of recurrence.^15^ Moreover, it is often challenging to effectively suture these large annulus fibrosus defects during surgery. Miller et al.^16^ reported in their meta-analysis that patients with large annulus fibrosus defects (≥6 mm) had a 2.5-fold higher risk of symptom recurrence and a 2.3-fold higher risk of reoperation than those with small defects (<4 mm) after lumbar discectomy. Patients with large annulus fibrosus defects are commonly seen in: (1) Severe extrusion- or sequestration-type LDH cases, where the annulus fibrosus is completely ruptured and significant nucleus pulposus protrusion results in severe tearing of the annulus fibrosus; (2) Complex cases where excessive resection of the annulus fibrosus during surgery leads to iatrogenic damage.^17^ Especially for elderly patients, compared to younger patients, the degeneration of intervertebral discs leads to reduced elasticity of the annulus fibrosus, thereby increasing the failure rate of annulus fibrosus suturing. In recent years, the exploration of new technologies for annulus fibrosus repair has become a focus. These innovations include mechanical closure devices, polymer material insertion, and pulsed radiofrequency thermal coagulation.^18^ Research into the biological regeneration of the annulus fibrosus via cell therapy, biomolecular therapy, and gene therapy is also gaining attention.^19^ These techniques not only repair the annulus fibrosus but also help delay disc degeneration.^20^ However, most of these methods remain in the animal experimentation stage, and substantial work is needed to translate them into clinical applications.

Our team innovatively adopted a technique of PLL coverage suturing repair for annulus fibrosus defects under full-endoscopic spinal surgery. This method involves repositioning the adjacent intact PLL to the area of the annulus fibrosus defect for suturing and repair. In this study, all patients were treated via the PEID approach, with no intraoperative vascular or neural injuries observed, reflecting the safety of this surgical method. Postoperatively, both groups showed significant improvements in the VAS score for low back pain and leg pain, as well as in the ODI score (P < 0.05). Compared with the preoperative values, the Pfirrmann grading at the final follow-up was significantly lower in both groups (P < 0.05). The postoperative recurrence rate in the observation group was significantly lower than that in the control group (P < 0.05), while there were no statistically significant differences in the perioperative condition between the two groups (P > 0.05). The study results indicate that this technique not only addresses the challenge of suturing larger annulus fibrosus defects during surgery but also effectively prevents reherniation of the nucleus pulposus, thereby reducing the recurrence rate. Although the surgical time for the observation group was slightly longer than that for the control group, the outcomes suggest that the observation group offers potential long-term benefits in improving surgical quality and patient satisfaction. Moreover, as surgical proficiency increases, the time difference is expected to decrease further.

However, this study has certain limitations. First, PLL coverage suturing in the area of the defective annulus fibrosus technique has a steep learning curve, requiring extensive training and experience accumulation to fully realize its safety and effectiveness. Second, the number of cases was limited, and the follow-up period was relatively short; therefore, the conclusions drawn from this study need to be further validated through larger sample sizes and longer-term follow-up studies.

## 5 Conclusions

In conclusion, employing full-endoscopic PLL coverage suturing in the area of the defective annulus fibrosus for treating LDH with annulus fibrosus defects yields satisfactory clinical outcomes. This technique effectively repairs annulus fibrosus defects and reduces the recurrence rate of LDH. This method represents a recommended and valuable surgical method worthy of broader application, particularly for patients with larger annulus fibrosus defects that are difficult to suture.

## CRediT authorship contribution statement

**Quan Zhang:** contributed to conception and design of the study, wrote the first draft of the manuscript, organized the database. **Bo He:** organized the database. **Zijin Liu:** wrote the first draft of the manuscript. **Zhenfei Wang:** wrote sections of the manuscript. **Jinhui Bu:** wrote sections of the manuscript. **Minghui Dong:** performed the statistical analysis. **Zhaobo Liu:** performed the statistical analysis. **Mengzi Hu:** wrote sections of the manuscript. **Chao Ma:** revised the manuscript. **Guangwang Liu:** contributed to conception and design of the study.

## Guardian/patient's consent

Consent for publication was obtained from the participants.

## Ethical statement

The studies involving human participants were reviewed and approved by the Ethics Committee of Xuzhou Central Hospital. The patients/participants provided their written informed consent to participate in this study. Written informed consent was obtained from the individual(s) for the publication of any potentially identifiable images or data included in this article.

## Funding statement

This study was supported by the Project of Xuzhou Plan of introducing a team of clinical medical experts (Grant No.2019TD002), Xuzhou Medical leading talents (Grant No. XWRCHT20210035), 10.13039/501100013059Jiangsu Provincial Medical Youth Talent (Grant No. QNRC2016392), the Scientific Research Projects of Jiangsu Provincial Health Commission (M2022048-LGY2018047), “Six one Projects” for High-level Health Talents in Jiangsu Province (LGY2018047), Jiangsu Province “333” talents pIan.

## Conflict of interest

The authors declare that the research was conducted in the absence of any commercial or financial relationships that could be construed as a potential conflict of interest.

## Abbreviations:

PLL: posterior longitudinal ligament
LDH: Lumbar disc herniation
VAS: Visual Analog Scale
ODI: Oswestry Disability Index
PEID: percutaneous endoscopic interlaminar discectomy
PETD: percutaneous endoscopic transforaminal discectomy
